# Medico-Chirurgical Transactions

**Published:** 1847-01-01

**Authors:** 


MEDICO-CHIRURGICAL TRANSACTIONS. Published by the Royal Medical and Chirurgical Society of London. Twenty-ninth Volume. Volume the Eleventh of the Second Series, 1846.

THE new volume of these Transactions has made its appearance this year in quite a new livery, being "got up" in a similar style to the volumes of the Sydenham Society, and with equal care and taste. The type, too, is of smaller size than in the former volumes, but is clear and legible. Although somewhat prejudiced in favour of handsome types and wide margins, we regard the change altogether as an improvement which we have no doubt will give satisfaction to the Fellows. Our business, however, is with matters of more importance, the contents, which we shall, as usual, examine in detail. But we must first notice the remarkable difference which exists in the quality of the Plates. The five by Basire, illustrative of Mr. Toynbee's Paper on the human kidney, are of great beauty, and superior to any we can recollect to have seen in former numbers of the Transactions. The first plate, also, by Bagg, is executed with the well-known skill of this artist, and will be admired for the distinct definition which the figures give of the objects they are intended to represent. These excellent plates put us quite out of conceit with the lithographic illustrations, which are indifferent specimens of the art, and inferior to those by Perry in former volumes. The Council, however, has been liberal in the supply of plates, which amount to eleven in number, and some of them are of large size, and contain numerous figures. The first paper excited a good deal of interest when the views of the author were announced to the profession. It is—

I. ON THE MINUTE ANATOMY AND PATHOLOGY OF BRIGHT'S DISEASE OF THE KIDNEY, AND ON THE RELATION OF THE RENAL DISEASE TO THOSE DISEASES OF THE LIVER, HEART, ARTERIES, WITH WHICH IT IS COMMONLY ASSOCIATED. By *George Johnson*, M.D.

Although our knowledge of the symptoms and effects of Bright's disease in the system is tolerably complete, pathologists are by no means agreed as to the precise nature of those changes which the kidney undergoes in the course of this disease. Dr. Johnson states that, he has "ascertained that the epithelial cells of the healthy kidney contain a minute quantity of oil, in the form of yellowish, highly refracting globules, the appearance of which in the cells of the liver is well known to microscopical observers. The quantity of fat in the cells of the healthy kidney is much less than in those of the healthy liver, but he has found it present, in greater or less quantities, in the healthy kidneys of more than twenty subjects which he has examined for this point, since his attention was first directed to it. Dr. Johnson does not seem to be aware that the presence of fat in the healthy kidney had been previously noticed by several of the German microscopical observers. He remarks—

"The secreting cells of the kidney, then, resemble those of the liver, in containing a certain proportion of oil, and the presence of this material in such a situation appears to indicate that a certain quantity of fat is excreted by the kidneys, as well as by the liver.

"Having premised thus much of the healthy epithelium, with its contents, Bright's disease may be described as *primarily and essentially an exaggeration of the fatty matter which exists naturally in small quantities in the epithelial cells of the healthy organ.*" P. 2.

A specimen of the disease in an advanced stage, examined with the microscope, presents epithelial cells in every degree of engorgement, from the incipient enlargement of the parietes in which the cell-nucleus is still visible, to the complete engorgement of the cells in which the nucleus is concealed by the fatty globules. The disease then appears to be a fatty degeneration of the kidney, precisely analogous to the fatty degeneration of the liver.

Dr. Johnson, before endeavouring to explain the changes which the kidneys undergo during the progress of Bright's disease, gives the following account of the arrangement of the vessels in the kidney as demonstrated by Mr. Bowman.

"A small terminal twig of the artery pierces the dilated extremity of the urinary tube: within the capsule thus formed by the expansion of the tube, the artery breaks up into a capillary plexus, which Mr. Bowman has named the *Malpighian tuft of capillaries;* these capillaries again unite into a single *efferent* vessel, which passes out through the capsule, and goes to form another plexus, which immediately *surrounds the urinary tubes.* The course of the circulation then is from the artery into the Malpighian plexus, which lies *within* the dilated extremity of the urinary tube, and from this plexus through the efferent vessel into the capillary plexus, which lies *external* to the tubes amidst their coils and convolutions." P. 5.

The author then adds :—

"Any one having a clear conception of the anatomy of the kidney cannot fail to perceive the effect upon the circulation through the gland, which must result from the changes which I have described as primarily and essentially constituting Bright's disease of the kidney. The fat accumulates in the epithelial cells to such an extent as to produce engorgement and dilatation of the cells, and of the tubes which are lined by them; the consequence is, compression of the capillary plexus surrounding the tubes, giving rise to congestion of the Malpighian plexus. This passive congestion of the Malpighian plexus leads to transudation of the serum of the blood, and sometimes to rupture of the delicate vessels of the plexus, and the consequent escape of the colouring matter and fibrin of the blood. These constituents of the blood pass into the tubes, and so become mixed with the urine. Their escape from the blood-vessels is the result of a mechanical impediment to the return of the blood consequent on compression of the veins by an accumulation of fat in the tubes." P. 6.

Dr. Johnson calls attention to the influence of a mechanical impediment in giving rise to the escape of serum and of blood, and their appearance in the urine, as shown by the ingenious experiments of Dr. Geo. Robinson, detailed in the 26th volume of the Society's Transactions. Dr. Robinson tied the renal vein in rabbits, the result of which was the appearance of albumen and of blood in the urine; the same consequences followed the partial or slow obliteration of the vein. Dr. Johnson notices that a mechanical obstacle to the circulation through the heart or lungs may give rise to venous congestion of the kidney and the consequent presence of serum and of blood in the urine without organic disease of the organ, and that the red spots visible on the surface of the kidney in some cases of Bright's disease, and which have been erroneously supposed to be dilated Malpighian bodies, were first shown by Mr. Bowman to be nothing else than the convolutions of a tube filled with blood, that has burst into it from the gorged Malpighian tuft at its extremity. The tortuous, dilated, and varicose condition of the veins and arteries, so often seen on the surface of the kidney, is likewise attributed to a mechanical obstruction.

Dr. Johnson notices a remarkable difference in the effect produced upon the two organs—the kidney and the liver, by an accumulation of fat in their substance; the function of the kidney becomes seriously interfered with, and its nutrition impaired, while the liver appears to suffer but little, either in its nutrition or its functions. We have not space for the account of the peculiarities of structure in the two organs, by which the difference is explained. The following remarks will be read with interest.

"Without anticipating what will presently be said of the pathology of Bright's disease, I will here offer a few remarks on its stages and forms; and, first, I will venture to assert that there is no inflammatory or congestive stage *preceding* the deposit. The congestion which often accompanies the disease, and which is a consequence of previous morbid changes, may be either active or passive. The way in which passive congestion occurs has already been sufficiently explained. Active congestion may be thus accounted for:—a large number of the epithelial cells become gorged with fat, and their secreting function is in consequence impaired: those portions of the gland which are less involved in the disease are now called upon to do an increased amount of work; this may lead to active congestion, and the consequent effusion of serum and blood into the tubes. In many cases there probably exists both active and passive congestion of the vessels; but I repeat that this is the *consequence*, and not the *cause*, of the deposit in the gland.

"Dismissing, then, a congestive stage, the earliest appearance of fatty degeneration of the kidney would, of course, be recognised by the aid of the microscope before the gland has undergone any change visible to the naked eye. As the accumulation of fat increases, the kidney becomes granular or 'mottled' on the surface. The smooth, mottled kidneys are such as have the greater number of the tubes in the cortical portion almost uniformly gorged; the gland is often much increased in size by the great amount of fat in the tubes, the vessels are much compressed, and the surface of the kidney sometimes presents an almost uniform yellowish white colour, with here and there a few vessels which have escaped obliteration. These are generally cases which have run a comparatively rapid course. The secreting function of the kidney has become greatly impaired, and death has been the consequence. The kidney which has arrived at this degree of fatty engorgement, probably never becomes atrophied.

"The *granular and atrophied* (Bright's) kidneys are those in which the accumulation of fat takes place less rapidly and uniformly; some convoluted tubes become gorged with fat, forming prominent granulations; and these, compressing surrounding parts, produce obliteration of the vessels and atrophy of the tubes, and thus the entire gland gradually wastes and contracts. These are the cases in which the tubes of the pyramids become filled with fat, part of which, perhaps, has been carried into them from above, while part is contained in their own epithelium, which perhaps, (as has already been suggested,) assumes a more active secretory office in consequence of the wasting of the cortical portion of the gland.

"I do not maintain that every atrophied kidney, and every kidney presenting a granular appearance, have undergone these changes in consequence of fatty degeneration of the gland; on the contrary, I am well aware that many instances of granular and contracted kidneys are met with, in which the degeneration has been of a totally different kind, and I am also in a position to show that these are not cases of true Bright's disease; that they belong to a class of diseases which the best pathologists have always endeavoured to distinguish from Bright's disease, although, in the absence of any accurate means of definition, diseases totally and essentially different in their nature have often been confounded under one name." P. 10.

Dr. Johnson's observations have led him to conclude that, in by far the greater number of cases, Bright's disease, or fatty degeneration of the kidney, is associated with a similar fatty degeneration of the liver. In the post mortem examination of 22 cases of Bright's disease of the kidney, there was in 17 a most marked degree of fatty degeneration of the liver. In four of the remaining five cases there was a decided increase of fat in the hepatic cells; and in one case only was there no such increase. During the period in which these cases were examined, Dr. Johnson met with only four cases of fatty liver which were *not* combined with Bright's disease of the kidney. In three of these cases, although there was not a decided fatty degeneration of the kidney, there was still a marked increase of fat in the epithelial cells of this gland. In the fourth case of fatty liver there was *no* increase of fat in the kidney. Of 23 cases in which both the liver and the kidneys might be considered *healthy*, there was an unusual quantity of fat in *both* organs in four cases, in the *liver alone* in two cases, in the kidney alone in two other cases; while, in the remaining 19 cases, the fat existed in what might be considered the usual quantity in *both* organs. It is needless to remark how important is a knowledge of these facts in any attempt to explain the pathology of Bright's disease.

"It has been fully established by the observations of Dr. Christison, MM. Solon and Rayer, and of many subsequent observers, that Bright's disease is very frequently associated with tubular disease of the lungs. Among the 49 cases from which my preceding observations are drawn, there were 14 in which tubercles were found in the lungs. Of these 14, there were six in which there was decided fatty degeneration of both the liver and the kidneys. In one case the *liver alone* was fatty, in one the *kidneys alône*, and, in the remaining 6 cases, there was either *no* increase of fat in the liver or kidneys, or the increase was not so great as to be considered morbid; so that, out of 21 cases of fatty degeneration of the liver, 6 only occurred in connection with tubercular disease of the lungs, while 17 occurred in combination with fatty degeneration of the kidney. The number of observations, I am aware, is too small to enable one to state exactly in what proportion of cases the different combinations to which I have alluded may be expected; but I feel assured that future observers will confirm my own conclusion that fatty degeneration of the liver in different degrees is more commonly associated with Bright's disease of the kidney than with tubercular disease of the lungs." P. 12.

According to Dr. Johnson's observations, it rarely happens that a patient dies of Bright's disease of the kidney without presenting more or less of that common change in the arteries which Mr. Gulliver has recently shewn to be a fatty degeneration of the vessels. A similar change has been observed in the valves of the heart. Although we have quoted largely from this interesting paper, we must find room for the following remarks.

"Let us now look back upon the ground we have gone over. We have seen that, in subjects who die of Bright's disease of the kidney, there is usually found a similar disease in the liver, and, in many cases, in the arteries and on the valves of the heart; the disease being in every case an increase and an accumulation of a material which exists in small quantities in the healthy condition of these parts.

"In any attempt to explain the *pathology* of these diseases, their source must be looked for in the process of digestion and assimilation. The processes of primary or secondary assimilation, or both, fail with regard to this fatty matter, which, not undergoing the changes requisite for its ready elimination from the system, or for its application to the nutrition of the tissues, is thrown into the circulation. An effort is made to carry it off by the liver and kidneys: the fat finds its way into the secreting cells of these glands; its escape from these parts, in a free state,is a slow and uncertain process, and, finding no material in sufficient quantity with which to pass off in a state of combination, the fat accumulates in, and obstructs, the glands.

"The increased amount of fat in the secreting cells of the glands must certainly be looked upon as an *effort* to carry off this material. It must also be looked upon as, in a great degree, an *unsuccessful effort.* It will presently be shown that the quantity of uncombined fat in the urine in cases of Bright's disease is seldom greatly increased. As far as regards the result of her effort, then, Nature is as unsuccessful in her attempt to carry off the fat by the glands as to remove it by throwing it into the arteries. In both cases the fat is thrown out of the circulation, but its accumulation in the glands and arteries leads to a serious interference with the functions of these parts.

"The conditions under which these diseases occur may be looked upon as analogous to those which give rise to diabetes. In diabetes, in consequence of imperfect digestion or mal-assimilation, sugar is eliminated in various excretions, but especially in that of the kidneys. Again, in the cases in which fatty degeneration of the liver and kidneys occurs, an effort is made to eliminate fat; the sugar being soluble, is readily carried off; the fat being insoluble, and consequently difficult of elimination, accumulates in the secreting cells of the glands." P. 15.

Our author calls attention to the fact, that Bright's disease is much more common in large towns than in the country, and in large towns is more prevalent among the intemperate, ill-fed, &c., than among those who enjoy more of the comforts of life. He mentions that Mr. Simon inspected the body of a cat, which had died after having been kept for about six weeks in a dark cellar. The kidney presented the appearance of a mottled Bright's kidney, and the tubes of the cortical portion were completely gorged with fat. The liver also contained an increase of fat, but in less amount than in the kidney.

Dr. Johnson, believing Bright's disease to be a constitutional disorder, next endeavours to show that acute inflammatory dropsy, which is commonly supposed to originate in this disease of the kidney, has really no necessary connection with it. He states that, exposure to wet and cold, and the consequent suspension of the cutaneous functions, may give rise to congestion of the kidneys, scanty albuminous and bloody urine and dropsy. But, "if the patient so attacked was previously of sound constitution, and if he be treated actively, with a view to restore the functions of the skin, to relieve congestion of the kidneys, and to carry off the accumulated fluid by the bowels, the dropsy and the other symptoms will disappear, and the patient will be restored to perfect health. Such an attack has no tendency to terminate in Bright's disease, which, it cannot be too often repeated, is not primarily a disease *in* the kidney, but a constitutional disease, manifesting itself *at* the kidney."

A similar view is taken of dropsy supervening upon scarlatina, which he considers as a disease essentially distinct from Bright's disease, being in fact an inflammation of the kidney which excites an increased development of the epithelium lining the urinary tubules. This material partly accumulates in, and chokes up, the tubes, while part of it becomes washed out with the urine, and may be detected in that fluid by the aid of the microscope.

The occasional occurrence of fat both in healthy urine and in Bright's disease is noticed by Dr. Johnson. He believes that epithelial cells containing fat are washed from the urinary tubes by the current of urine, and he looks upon their presence in the urine as one of the most certain signs of the existence of Bright's disease—especially of its early stage, as at this period a greater quantity of oil escapes than at a later stage of the disease, when the tubes are more uniformly choked by their accumulated contents.

Not much is said on the subject of treatment. As would be expected from the view taken by the author, of the renal disease being a local manifestation of a general constitutional disorder, the remedies recommended are chiefly those of a hygienic character, accompanied with a caution to avoid a fat diet, and to be moderate in the use of such materials as starch and sugar, which seem difficult of digestion, and which may, perhaps, by a slight chemical change, be converted into fat. Local bleeding may sometimes be required for the relief of the congested condition of the kidney and is often followed by great benefit, but it must be used with caution. Dr. Johnson, in conclusion, states that he hopes to have established the following points :—

"1. That the epithelial or secreting cells of the healthy kidney contain a certain quantity of oil; the proportion of which, under certain circumstances, and within certain limits, may fluctuate considerably.

"2. That it is an excessive increase of this fat, leading to engorgement of the epithelial cells, and of the urinary tubes, which constitutes primarily and essentially Bright's disease of the kidney.

"3. That the presence of albumen and blood in the urine, and the wasting of the tissues of the kidney, are secondary phenomena, dependent on the mechanical pressure of the accumulated fat.

"4. That in the majority of cases, Bright's disease is associated with a similar fatty degeneration of the liver and arteries, and frequently of the valves of the heart; these diseases being related to each other as joint effects of one common constitutional cause.

"5. That probably acute inflammatory dropsy, occurring in a person previously healthy, and the dropsy which occasionally supervenes upon scarlatina, have no necessary connection with Bright's disease of the kidney.

"6. That most important evidence of the approach and presence of the renal disease may often be derived from a microscopical examination of the urine, in which will be found fat in unusual quantity; partly in the form of free oil-globules, and partly contained in epithelial cells which have escaped from the urinary tubes.

"7. That the insight which we have obtained into the peculiar change which the kidney undergoes in Bright's disease, and the knowledge we possess of the simultaneous occurrence of a similar change in other organs, may serve as important guides in the prevention and cure of the disease." P. 22.

This paper affords evidence of no ordinary ingenuity and labour, and it cannot fail to raise Dr. Johnson's reputation as a minute pathologist. We have not attempted more than to furnish an abridged acount of the results of our author's researches, though well aware that some of his conclusions have been objected to, and that his views generally have been regarded as too exclusive. In order to present to our readers a connected account of the most recent opinions on the pathology of Bright's disease, we shall now proceed to analyze two other papers on this subject, contained in the Transactions, though not in the order of succession.

II. Account of a Case of Congenital Deficiency of one Kidney with Granular Degeneration of the Existing One. By *George Busk*, Surgeon to the Seaman's Hospital Ship "Dreadnought."

The patient, a gentleman, about three years before his death, which occurred at the age of 27, became bloated and pale, and incapable of as much exertion as formerly. He continued, however, his usual pursuits up to Christmas last. On the advice of a friend he took a quantity of calomel so as to produce ptyalism, and in March he consulted Mr. Ceely of Aylesbury.

"At that time the bowels were occasionally irritable, with an inordinate secretion of bile, and sometimes torpid, with a deficiency in that secretion. His appetite was lost, and he complained much of languor and lassitude; his legs also became anasarcous. He suffered from occasional attacks of painful swelling of the foot, which were of short duration, and considered to be of a gouty nature. The stomach became more and more irritable, and he frequently vomited. He was troubled with epistaxis to a great extent, and, it appears, had been subject to nasal hæmorrhage from childhood. In May, on the occasion of an attack of the gouty affection, the urine is noted to have been acid and albuminous, with a specific gravity of 10·10. He passed about three pints in the 24 hours. The superficial veins on the surface of the chest were at this time (May 5th) observed to be considerably enlarged. The heart's sounds are stated to have been normal, but there was dulness, on percussion, over a considerable space in the præcordial region, and the impulse of the heart is described as inordinate; the pulse 104. Mr. Ceeley noticed, on the subsidence of the ptyalism, a small crack-like sore on the frænum linguæ, which appears never to have healed; from the 3rd to the 6th of May there was constant oozing of blood from the mouth. On the 10th of May, to the other symptoms was superadded a painful attack of hæmorrhoids. The tongue was, towards the end of life, extremely sore, and cracked longitudinally; the urine pale, and reduced in quantity to one pint and a half in 24 hours, and highly albuminous. The hæmorrhage from the mouth and nose frequently recurred, and latterly was copious, and almost continuous. The strength became much reduced, especially in the lower extremities, where the weakness amounted to a degree of paralysis. A few days before death, the under side of the tongue and the inner surface of the cheeks became gangrenous, and the mouth afforded a copious viscid, mucoid and extremely offensive secretion. The body exhaled a strong and peculiar, and somewhat urinous, odour. Death took place on the 16th of May." P. 271.

The body was inspected by Mr. Busk 24 hours after death. It retained the strong urinous odour. The following were the chief morbid appearances. The right pleural cavity contained about six ounces of clear serum.

The left was obliterated by old adhesions. The pericardium occupied a considerable space, and contained about four ounces of serum, in which abundance of urea was detected. The heart was also double the natural volume and loaded with fat externally. The cavities were all equally enlarged, and the left ventricle was hypertrophied. The valves were healthy. The omentum and mesentery were loaded with fat. The liver was about one-third larger than natural. It did not grease the scalpel, but under the microscope it presented a large quantity of oil. No trace whatever of the left kidney or supra-renal capsule could be found on the most careful search. "The right kidney was about 2 inches in length, by $1\frac{1}{4}$ in width, and presented externally a contracted or corrugated aspect, and the roughened capsule could not be stripped off to any extent without laceration of the substance of the gland. The colour of the kidney was very pale, speckled with white, and on a section, scarcely any distinction of substances was observable. The whole presented a condensed, cicatriform aspect, of a semi-transparent hue, sprinkled with small opaque specks, and, in the remains of the tubular portions, marked with white striæ, obviously vessels filled with a dense white material The pelvis was remarkably small, and the mammillary processes appeared also to be unusually few in number." The left ureter entered the bladder at the usual situation, and was traced upwards in the natural course for about six inches, where it ended in a cœcal point. The tube was very narrow but pervious to the bladder.

"The substance of the kidney was examined microscopically: the tubuli uriniferi were found to be in parts indistinct, or obliterated, and in others to be filled with a semi-opaque white granular material, soluble, or rendered transparent by acetic acid, and presenting none of the characters of oil, very few globules of which were observed in any part of the gland. It was the dense white material, consisting of minute sub-globular refracting particles, soluble in acetic acid, the accumulation of which in the larger straight tubuli uriniferi constituted the white striæ above-mentioned." P. 273.

Mr. Busk remarks that, on referring to the particulars of the numerous cases of the total absence of one kidney on record, it will be observed that, in the majority of them, the deficiency of one gland has been compensated for, by an increased size of the existing one, or that the existing kidney presented, in addition to its abnormal bulk, sufficient evidence, in its irregular form, of its being constituted by the coalescence of two. Other cases, again, are mentioned, in which the size of the existing gland has not exceeded the natural standard, among which the present case is to be classed. "In this respect, then, it presents nothing new, and there is every reason for supposing, that had the existing kidney retained its healthy condition, good health might have been enjoyed with one kidney, rather below the average size. But the present instance shows more particularly what a very small portion indeed of secreting structure in the kidney is compatible with tolerable health." We fully coincide in this observation. As to the prediposing causes of the disease in the kidney, Mr. Busk observes that, unless the condition of the heart be considered as such, there was no other obvious cause in this case, excepting the double duty the gland was called upon to perform.

"The pathological condition of the kidney, and the presence of albumen in the urine, are clearly, in this case, not to be referred to the secretion and deposition of oily matter in the tubuli uriniferi. It affords, on the contrary, an instance of what may probably be considered the effects of chronic adhesive inflammation of the venous plexus and tubuli uriniferi, causing partial obliteration of the former, and contraction and obliteration of the latter, or their infarction with solid albuminous matter. The latter phenomenon is one so frequently met with in various affections—such as scarlatina, jaundice, and active congestion or inflammation of the kidney from exposure to cold, &c.—that it might, perhaps, be readily admitted as a frequent cause of degeneration of the gland, and which I believe it is. The adhesive capillary phlebitis, which I presume to have been the principal cause of the contraction and destruction of the glandular tissue, is so closely analogous to what occurs in other organs, especially the liver, and which ultimately produces in them cicatriform contraction and induration, that the probable identity of the affection in all these cases may fairly be assumed." P. 274.

Mr. Busk attributes the obliteration of the capillary venous plexus to the formation in those vessels of fibrous clots, such as are met with in certain circumstances in nearly every part of the venous system. The examination of a great many kidneys affected with granular degeneration, as occurring in the active class of men who come under observation in the Seaman's Hospital, has led him to conclude that adhesive inflammation of the tubuli uriniferi and venous plexus of the kidney, is, among that class, by far the most frequent cause of chronic albuminuria, and what is termed granular degeneration of the kidney. And he thinks that "the presence of oil in the tubuli uriniferi, though undoubtedly of frequent occurrence, has no direct or necessary influence in the production of albuminuria, for the reason that such an undue secretion of oil by the kidney may exist to a very great extent, without any albumen being present in the urine, as may be observed in certain cases of jaundice for instance; and on the other hand, because albuminuria may exist, and all the phenomena of suppressed secretion of urea be produced, without any oil being discernible in the tubuli uriniferi."

Mr. Busk is also inclined to believe that the deposition of oil in, or in other words, its secretion by, the kidney, is, in most cases, concomitant with some affection of the liver, by which, the special function of that gland being impeded, the kidney acts, as it were, vicariously, and eliminates some of the carbonaceous matter which should have been eliminated by the liver in the form of oil, &c. That the kidney, in case of jaundice, assumes this vicarious action, is sufficiently obvious, and that the bile is actually secreted in the epithelial cells of the tubuli uriniferi, may also be distinctly seen. In other cases, however, the cause of the kidney secreting oil may be traced, perhaps, to pulmonary disease, and in these cases also it is to be looked upon as a vicarious action.

This is a case of considerable interest. Had the patient possessed two kidneys instead of a single one, his life would in all probability have been longer preserved, and the important functions of these organs would have continued to be performed sufficiently for the purposes of life to a later period even if both kidneys had become implicated in disease. It will be noticed that our author's views of Bright's disease differs materially from those of Dr. Johnson, in so far as Mr. Busk considers adhesive inflammation of the tubuli uriniferi and venous plexuses of the kidneys as a common cause of the affection, independently of any fatty deposit. The presence of albumen in the urine may, however, be due, as supposed by Dr. Johnson, to a mechanical cause, the fibrinous matter producing a similar effect on the circulation of the gland to that attributed to the fatty deposit. Mr. Busk's observations, however, do not imply that a structural change in the kidney and albuminous urine may not arise from the deposition of oily matter in the tubuli uriniferi, but that the latter is not the invariable, or the most frequent, cause of Bright's disease. The class to which this case belongs, we presume to be that which Dr. Johnson has announced his intention of showing to be essentially distinct from Bright's disease.

III. ON THE INTIMATE STRUCTURE OF THE HUMAN KIDNEY, AND ON THE CHANGES WHICH ITS SEVERAL COMPONENT PARTS UNDERGO IN BRIGHT'S DISEASE. By *Joseph Toynbee*, F.R.S. &c.

Mr. Toynbee informs us, in a note at the commencement of this paper, that he has for three years, in conjunction with Dr. Bright, been conducting investigations into the intimate structure of the kidney, but that various circumstances arose to prevent the issue of a work which had been designed to comprise the results of their joint labours. We know not what these circumstances were, but we suspect that, in most points of importance in respect to the minute structure of the kidney, they were anticipated by Mr. Bowman, in his admirable paper which appeared in the Philosophical Transactions for 1842. Mr. Toynbee states that he is now indebted to the kind liberality of Dr. Bright for the opportunity of using the drawings and engravings taken from the preparations at his expense, and generously adds, that nearly everything of value which may be contained in the observations which follow is to be attributed to the assistance of Dr. Bright, and that it is not without some diffidence that he (Mr. T.) ventures to prefix his name to the present communication.

The publication of the fruits of these investigations, whether confirmatory or opposed to the views of Mr. Bowman, cannot fail to prove of interest to the profession, and the descriptive part, in conjunction with the beautiful engravings, form a valuable contribution to the Society's Transactions. We fear that we cannot convey to our readers in an abridged form and without the illustrations a clear view of the minute anatomical structure of the kidney, an account of which forms the bulk of this paper. Those who are interested in the subject will no doubt consult the volume itself, and we must be content with noticing one or two points in which the writer differs from Mr. Bowman. The latter anatomist asserts that the corpora Malpighiana cannot be injected from the tubes. Mr. Toynbee has many specimens in which these bodies have been so injected, and he adduces the authority of Dr. Gerlach, of Mayence, in confirmation of his statement. This anatomist has also succeeded in injecting the tubuli uriniferi from the ureter and the Malpighian capsules at the same time. Mr. Toynbee is also at variance with Mr. Bowman in agreeing with those anatomists who state that the tubuli uriniferi terminate in a plexiform manner by communicating with each other, which Mr. B. denies, and believes to be founded on deceptive appearances. Mr. Bowman describes the tubuli as having an expanded origin, the expansion being composed of the basement membrane of the tube and enclosing in it the wounded tufts of capillary vessels usually designated the corpora Malpighiana. Several modern anatomists have doubted the accuracy of this view, and Mr. Toynbee is also opposed to it. He states that the capsule of the corpus Malpighianum, instead of being, as supposed, an expansion of the tubuli, is a distinct globular investment, enveloping both the tubuli and the tuft of vessels. This globular investment is neither continuous with the tubuli, nor with the blood-vessels, but is expanded over them. Into one part of the capsule the artery enters, while the other receives the tubuli. The artery divides and subdivides, so as to form a globular mass of capillaries in the interior of the capsule from which the efferent vessel emerges. The tubuli, after penetrating the capsule, becomes tortuous, and twists into a coil, and after being in contact with the ramifications of the corpus, it emerges from the capsule. There are some minor points of difference in respect to anatomical detail to which, if our space permitted, we should be induced to allude. We can only add, in concluding our notice of this part of the paper, that Mr. Toynbee's observations, being the result of careful research and much labour, and tending to correct some erroneous views, are well deserving of record, but we are of opinion that they do not contribute materially to our present knowledge of the minute structure of the kidney.

Mr. Toynbee, after remarking " there appears to be no doubt that the true cause of this disease is the circulation of the blood of the organ, of an unnaturally large quantity of carbonized and azotized elements," and that it has been proved that the ultimate effect of this supercarbonized state of the blood is the deposition, in the kidney, of adipose matter, plainly gives in his adhesion generally to the views of Dr. Johnson, whose paper appears to Mr. Toynbee to be the only account which exhibits the disease in its true relations. He differs, however, from Dr. J. in one important point, viz. in believing that a state of congestion precedes any structural alteration in the kidney, so far agreeing with Dr. G. Robinson, Mr. Busk, and others. Our author remarks :—

" Now, considering that in this disease the blood is highly charged with carbonized principles, and, consequently, that in its circulation through the kidney, that organ must be called upon to throw off a larger quantity of carbonaceous matter than the natural secretion would contain, an amount of irritation will be excited which must be followed by nervous depression and ultimate congestion of the entire organ. This general view, combined with the results of my investigations into the early stages of the disease, induces me to agree with Dr. Bright and others, that the congestive condition of the blood-vessels of the organ does precede, and that necessarily, the deposition of fat, the enlargement of the organ itself, or of its uriniferous tubes, or of any other of its vessels.

" The cause of the presence of albumen in the urine is acknowledged to be an obstructed condition of the blood-vessels of the organ. Dr. Johnson considers the obstruction to arise from a deposition of fat in the tubuli uriniferi; but there can be no doubt that albuminous urine often exists without any such deposition." P. 320.

Mr. Toynbee divides Bright's disease into three stages, each of which is founded on certain pathological conditions of the organ ; but antecedent to the development of any of these changes, he believes that the organ is for some time in a state of congestion. In the first stage, "the kidney is enlarged, and innumerable black points are visible, which are the corpora Malpighiana dilated, and their vessels distended with blood, seen through the capsule. The white spots, which derive their appearance from the collection of fatty matter, begin to be perceptible.

"The peculiar features of this stage consist of an enlargement of the arteries entering the corpora Malpighiana; the dilatation of the vessels of the tuft, the capillaries and the veins; an increase in the size of the capsule of the corpus and of the tubuli, and a large addition to the quantity of the parenchyma of the organ." The artery and the corpus is twice or thrice its natural size, which is the case also in the Malpighian tuft and the capillary vessels which spring from the tuft. The capillaries and veins are greatly enlarged, giving to the surface of the organ the resemblance of network. This is the commencement of the stellated condition which is so marked a characteristic of the next stage of the complaint. The tubuli are also much increased in their dimensions; but the fat which is found in them is soft and white.

In the second stage, the organ is very greatly increased in size, its surface is smooth, and presents numerous white spots; the capsule is but slightly adherent to the surface and the tissue of the organ is flabby. The structural changes exhibited during this stage are the following.

"The artery of the corpus Malpighianum becomes so greatly enlarged, that frequently it equals the dimensions of the tube itself, and is eight or ten times its natural size. It is tortuous and dilated, and sometimes, previous to entering the capsule of the corpus, presents analogous swellings to those of varicose veins. The primary branches of it, in forming the tuft, are also distended to ten or fifteen times their natural size, and are not unfrequently discovered external to the capsule of the corpus, as though thrust out by some external force. The vessels forming the tuft are likewise enormously enlarged, and very often the minutest branches are fully as large as the main artery of the corpus in a healthy state.

"Occasionally the tuft is broken up, and instead of forming a compact mass, exhibits its individual branches separated from each other. At other times the branches of the tuft are actually larger than the primitive artery of the corpus." P. 321.

Mr. Toynbee is surprised at Mr. Bowman's statement that he has never seen, in any one instance, a clearly dilated condition of the Malpighian tuft of vessels, which is attributed to the peculiar injection used by that anatomist. An enlargement of the renal arteries and dilatation of their branches are also observable in this stage of the disorder. The capsule too is greatly increased in size.

"The tubuli differ considerably from their healthy condition, being enlarged to two or three times their natural size, and aggregated together in masses, so as to lie in contact with each other, and form definite, roundish bodies: they are also extremely convoluted with numerous dilatations; frequently they are varicose. At other times they present distinct aneurismal sacs, which bulge out from one part of the wall of the tube, to which they are attached by a small neck or pedicle. Occasionally, some of the vessels of a convolution are smaller than the others, and their size nearly natural. The tubuli in the masses are so closely packed, that the blood-vessels are evidently compressed, and rendered incapable of admitting an injection. At times a tube, even at some distance from the corpus, becomes very convoluted, and knotted into a mass." P. 322-3.

In cases where the kidney is much enlarged, the parenchymatous cells will be found not merely increased in size, but adipose depositions will be visible throughout them.

In the third stage of the disease " the kidneys are smaller than their natural size ; hard, white granules are prominent on their surface, which is more or less lobulated ; the capsule is adherent ; vesicles of large size are frequently everywhere interspersed ; and numbers of smaller ones stud the whole surface. On making a section, the organ is found to be deprived of blood ; the cortical part contracted, the blood-vessels large, and their walls thick."

The arteries are in a more contracted condition than that described in the second stage ; and the Malpighian tuft is so often changed from its natural state, that the greater part of its vessels are not capable of being injected. The capsule of the corpus has assumed a more contracted appearance. " The tubuli are larger than in the preceding stage, and are gathered into rounded masses, which form the granules on the surface of the organ. The latter are of a white hue, and are most commonly fully distended with fatty depositions ; though not unfrequently they appear like dark spots : the tubuli in that case being full of blood. A rounded appearance is generally characteristic of the granules, in each of which the component tubule forms innumerable convolutions." The tubuli are filled with oily cells, granular matter particles of various size, and blood globules. The parenchyma is hard, and is composed of elongated stellated cells, from the angles of which fine threads proceed, and communicate with each other. There is nothing said on the subject of treatment. Mr. Toynbee agrees with Dr. Johnson in recommending hygienic measures in order to prevent the development of the disease.

We must confess that this paper has disappointed us, especially as great pains have confessedly been bestowed on the subject of which it treats. There is less of novelty and interest in the pathological part even than in the anatomical, and no conclusions of a practical character are deduced from the author's researches.

### IV. History of a Case of Ligature of the Left Subclavian Artery between the Scaleni Muscles, attended with some Peculiar Circumstances. By *J. C. Warren*, M.D, Professor of Anatomy and Surgery in Boston, U. S. A., Honorary Fellow of the Royal Medical and Chirurgical Society, &c.

Dr. Warren remarks that the history of an operation for the ligature of the subclavian artery would seem scarcely worthy the attention of the Society. This operation has been done many times in various parts of the world, and the annals of this distinguished body contains no less than twelve cases. The case which he has the honor to lay before them possesses peculiarities, and will, he hopes, afford some practical inferences.

James Avery, aged about 30, on the evening of Dec. 23rd, 1843, while in a state of intoxication, slipped on the ice, fell, and struck his left shoulder against the curb-stone of the side walk. Surgical aid was called, and violent efforts were made to reduce the dislocation, but in what manner the patient could not tell, excepting that he thought one person placed his foot, with a boot on, in the axilla. He was sent to the hospital, and on the next day was seen by the author, who found the left arm and shoulder much swollen. Leeches and cold applications were employed, and on the following day the swelling was so much reduced as to enable him to decide that no dislocation existed. During the night of the third day following, Dec. 28th, the patient was seized with a violent fit of coughing, in which he felt something give way in his shoulder. The next morning the shoulder and arm were very much discoloured and enlarged, the arm was painful and the patient much prostrated. On the 30th, it was discovered that the man had no pulse in his left wrist or in any part of the arm, and he had also lost both feeling and motion in the extremity. The swelling increased until it became enormous, the arm turning black in the axilla. A vesication was noticed on the back of the fore-arm. January 27th, 1844, an abscess was found to be forming in the axilla. In seven days it pointed, but did not open till Feb. 4th, when it discharged a coagulum and about a pint of fluid dark-coloured blood. Three days subsequently, at six o'clock in the morning, a sudden gush took place from the wound, by which the bed was inundated, the mattresses soaked, and blood poured upon the floor. Exhausted and almost lifeless he sunk into a state of syncope and the hæmorrhage ceased. As he was too low to undergo any operation, it was agreed that, if he lived to the next day, the subclavian should, if possible, be tied. By the next morning, he had much revived. At ten o'clock he took eighty drops of the tincture of opium, and at eleven was carried into the operating theatre.

A great difficulty presented itself in the outset of the operation, the swelling of the shoulder, the tumour in the axilla, and the natural shortness of the neck, almost obliterating the space between the shoulder and lower jaw. Dr. Warren, after minutely detailing the steps of the operation, states, that the aneurism-needle was passed under the first dorsal nerve which was mistaken for the artery. The wound was too deep, too narrow, and of consequence too dark, to permit the artery to be visible. The anterior scalenus was partially visible, and, passing the fore-finger of the left hand to the edge of this, a good portion of the muscle was divided by the probe-pointed bistoury, introduced upon the finger. The subclavian artery then became quite sensible to the touch, and slightly distinguishable by the eye. A long aneurism-needle was passed under the artery, and at this moment a slight whistling was heard, and the author was satisfied that some air had entered the thorax. The ligature was tied, and the wound closed.

The patient improved after the operation. On Feb. 22nd, the thirteenth day, the ligature was removed. On the 29th, a stream of blood was seen to issue from the unclosed part of the wound: the blood lost amounted to about a pint, did not issue per saltum, and was of a venous colour. The hæmorrhage was arrested by pressure. At the commencement of March he had an attack of pneumonia confined to the lower lobe of the left lung, and also a second attack about the 1st of May. By the 1st of October the swelling had disappeared from the arm, and motion had returned in the shoulder-joint. The large excavation in the axilla was reduced to a fistulous tube. On Feb. 4th, 361 days after the operation, the author was able for the first time to detect a distinct pulsation in the radial artery, and subsequently one of an indistinct character in the ulnar and brachial. The patient, June 15th, had nearly recovered. There were still fistulous openings in the neck, and axilla. Sensation and motion were slowly improving.

The author remarks that the cause of the rupture of the subclavian artery in this case is involved in some obscurity. The probability seems to be, that great violence was employed in the reduction of the dislocated humerus, and that the arteries and nerves were contused by strong pressure of the operator's boot, combined with the forcible extension of the arm. The vessel did not rupture immediately, because its coats were contused and not torn asunder, but a separation of the contused parts took place, in consequence of the violent efforts of coughing on the fifth day after the accident.

The detailed account of this case is somewhat prolix. It is however a remarkable instance of recovery from great perils, and the favorable result was mainly due to the skill and care of the Doctor.

V. Two Cases of Disease of the Brain, following the Application of a Ligature to the Carotid Artery. By *John P. Vincent*, Esq. Surgeon to St. Bartholomew's Hospital.

Mr. Vincent states that the two following cases appear to possess an interest that entitles them to be placed under the consideration of the Society.

James Mason, æt. 48, in July, 1829, was admitted into St. Bartholomew's Hospital with an aneurismal tumour under his right ear. It had been forming eight months, and was about the size of a small orange. On July 18th, Mr. Vincent tied the common carotid artery. In about an hour and a half after the operation the patient was discovered to be slightly convulsed on the right side. He afterwards sunk into a state of stupor. He was bled to ℥xxx. After this he became more sensible. He had twitchings of the right side. He was again bled during this and the two succeeding days, and altogether lost ℥84. His left side became paralysed, his urine and fæces passed off involuntarily, he swallowed with difficulty, and on the 24th he died. On examination of the body, it appeared that the veins of the right side of the brain were not so filled as those on the left. The substance of the brain on the right side was quite soft and cream-like.

On the 9th of April, 1845, William Brown, aged 28, was admitted into the Hospital, having stumbled a few hours previously against a door whilst smoking a pipe, which penetrated the tongue anterior to the right tonsil. The patient felt a portion of the pipe in the wound, but was not able to remove it. There was some bleeding, which gradually subsided. His voice was husky. He complained of pain, which was encreased on his attempting to swallow or to open his mouth. On introducing a probe into the wound no foreign body could be detected. The parts around the wound were greatly swollen. During the next five days the swelling gradually increased, and impeded deglutition. On the morning of the 5th day his breathing became difficult. On the 16th, hæmorrhage took place to the extent of ℥xxiv., which the house-surgeon arrested by pressure. Mr. Vincent was sent for, and fearing that, if the pressure was remitted, the hæmorrhage would proved fatal, he proceeded to tie the carotid artery. It was observed during the operation that the patient made violent efforts with his right side, but that he never moved the left extremities. During the night the left extremities were frequently convulsed. His pulse, which had been 132, sunk to 96. During the next two days the twitchings of the right side and paralysis of the left side continued. About midnight of the 18th, whilst coughing, about an ounce of arterial blood flowed through the nose and mouth and from the wound in the neck. On the 21st, a fit of coughing, with hæmorrhage to the extent of two or three ounces from the nose and mouth, terminated the patient's existence.

*Examination of the Body.*—The wound in the neck presented a sloughy appearance. At the bifurcation of the carotid on the right side there was a large and firm coagulum, in the middle of which was the extremity of the tobacco-pipe which had penetrated the artery at the point of division into external and internal carotids. The convolutions of the brain on the right side were flattened and softened. On dissecting the brain, irregularly-shaped cavities filled with ash-coloured effusion and shreds and particles of a greenish hue were discovered.

Mr. Vincent adds, what must be obvious, " that if the portion of the tobacco-pipe had been detected at the time the patient was brought to the hospital, and withdrawn from its position in the wound, he must have died instantly, from the gush of blood that would have taken place, as this body seemed to have completely plugged up the artery. I understand that such a sudden fatal event has occurred under similar circumstances."

These two interesting cases will draw the attention of surgeons to a source of danger from the operation of tying the carotid artery, which has been in a great measure overlooked. Dr. Norman Chevers, in an elaborate paper, entitled, Remarks on the Effects of Obliteration of the Carotid Arteries upon the Cerebral Circulation, published only last year in the Medical Gazette,* has particularly noticed the erroneous opinions which prevail on this subject, and has collected no less than twelve cases in which cerebral disease, and in ten instances death, resulted after one of the carotid arteries had been tied. He states, also, that he has perused nearly all the recorded cases in which the carotid artery has been tied, and found that, in many of the patients who did not die of brain disease, cerebral symptoms of a very decided kind occurred, such as drowsiness, giddiness, dizziness, delirium, paralysis of the corresponding side of the face, severe pain in the head, alarming faintness, vertigo, &c. Indeed, in most of the cases, some one or more of these symptoms has presented itself, yet the writings of Mr. Samuel Cooper, Sir A. Cooper, M. Manec, Mr. James Miller, and others, on the operation, tend to shew or directly affirm, that the carotid may be tied without risk of injury to the brain.

---

* Vol. 36, October 1845.

VI. Case of Punctured Wound and Ligature of the Posterior Tibial Artery, in the Upper Third of its Course. By *James Moncrieff Arnott*, F.R.S. Surgeon to the Middlesex Hospital.

Mr. Arnott remarks that, when an arterial trunk or main branch is wounded, the rule of practice is that the vessel should be tied at the seat of injury, or as it may be more truly expressed, when it can, it ought to be tied at the seat of injury, as the most certain means of arresting hæmorrhage; the qualification being rendered necessary by the fact that it is sometimes impossible, or is deemed inexpedient, to put the principle into practice. The circumstance which chiefly operates in creating an exception to the rule is the situation of the wounded artery; and it is this no doubt which has influenced those who, in punctured or gun-shot wounds of the posterior tibial artery high up, have recommended other means of meeting the difficulties and dangers of the case.

A case is adduced of punctured wound of the posterior tibial high up, where Sir Astley Cooper tied the femoral artery, but the bleeding recurred, and the limb was amputated, the patient not surviving. In future cases of the kind Sir A. Cooper advises immediate amputation. Mr. Arnott mentions another case of wound of this artery by a ball, in which M. Dupuytren tied the femoral artery, and the patient recovered. Mons. D. in consequence recommended that, when the principle artery of a limb ruptured by a ball is followed by an aneurismal tumour, the vessel should be tied between it and the heart. On the other hand, Mr. Guthrie severely criticises this eminent French surgeon. He relates two cases of gun-shot wound of the leg attended with hæmorrhage, in both of which the femoral artery was tied, but the bleeding recurred, leading to amputation, and death. In another instance Mr. G. cut boldly through the muscles of the calf, and tied the Peroneal artery, which had been wounded, thereby saving the limb—and he recommended that a similar proceeding should be adopted when the posterior tibial is wounded.

Mr. Arnott notices, in addition to this difference of opinion as to the treatment to be pursued in wounds of the posterior tibial, another which exists as to the mode of reaching it high up in cases of aneurism or secondary hæmorrhage lower down;—some surgeons recommending the division of the whole thickness of the muscles of the calf; others, an incision on the inner edge of the tibia, the gastrocnemius being pulled aside and the soleus alone divided. That ligature of the femoral artery is an uncertain means of arresting hæmorrhage from the posterior tibial, is shown by the three cases which have been adverted to, and as Mr. Guthrie's proposal of cutting directly through the muscles of the calf has not hitherto been put into practice, Mr. Arnott thinks the following case possesses claims upon the attention of the Society.

A robust young man was admitted into the Middlesex Hospital on January 1, 1845, with a punctured wound from a joiner's chisel in the calf at the junction of the upper with the middle third of the leg, and a little to the inner side of the mesial line—arterial and venous blood flowed in quantity. From the situation, depth, and direction of the wound, as ascertained by a probe, it was evident that the posterior tibial was probably wounded. Mr. Arnott determined to cut down upon it at once, in order to secure both ends. He made an incision through the skin and muscles of the calf, to the extent of 6½ inches; the deep fascia being thereby exposed, the opening in it made by the chisel was enlarged to the extent of two inches. After considerable difficulty from the bleeding, it was ascertained that, besides the wound in the posterior tibial, both venæ comites were divided. In consequence of the troublesome character of the bleeding from these veins, and the difficulty created in discovering the artery, one of them had a ligature placed on both ends, whilst the lower end of the other was subjected to pressure. Two ligatures were then placed on the artery, one above the other, below the puncture—it was not till the latter was tied that the hæmorrhage ceased. But little febrile disturbance followed the operation; the lower ligature on the artery came away on the 8th day—the upper on the 9th. During the night of the 11th some bleeding took place from the lower angle of the wound, which was not arrested by compression of the femoral artery, but which was easily checked by displacing some coagula from the wound, and making pressure at the lower part of it by means of a small compress of lint, which was left in the wound;—this was removed in three days, and the case proceeded subsequently uninterruptedly to a favorable termination. The wound cicatrised in less than two months, and the patient recovered with the limb as efficient as the other.

Mr. Arnott remarks, that the incisions in this instance were wholly within the limits of the calf, and that the pain and spasm of the divided muscles from pressure, in separating the sides of the wound during the operation, were considerable; but the venous hæmorrhage chiefly tended to render the operation tedious and difficult. He states—

"Taking all circumstances into consideration, it must be allowed that the operation of cutting down upon the posterior tibial artery when wounded under the calf, and tying it, is an operation severe to the patient, and troublesome to the surgeon, requiring both time and patience on his part. On the other hand, when we consider that the object of this operation is to save limb and life; that by adopting it we take the most likely method of attaining these objects, by putting in practice the most certain means of arresting arterial hæmorrhage; that upon the evidence afforded by this case, no danger arises from the mere size of the wound, and no permanent detriment from the extensive division of the muscles,—I think it must be conceded, until experience shows the contrary, that this is the proceeding which ought to be adopted in wounds of this artery high up." P. 51.

A case of wounded posterior tibial artery which occurred to Mr. Lawrence is related, in order to shew the importance of early interference, and the injurious effects of internal extravasation. Mr. Arnott, in conclusion, ventures his "opinion, that when the posterior tibial artery requires to be tied high up, on account of aneurism lower down, the easiest mode of reaching it will be by cutting directly through the muscles of the calf, instead of by incision at the edge of the tibia."

This is a paper of considerable practical value. The facts may not be sufficient to establish the proceeding recommended by Mr. Arnott as a rule of practice, but they show that our present experience is in favour of such a course.

Most of the papers in this volume are of great interest, and we shall return to it in our next number.

